# Stretching the limits of submucosal tunneling endoscopic resection

**DOI:** 10.1055/a-2233-3327

**Published:** 2024-01-30

**Authors:** Diogo Bernardo Moura, Nuno Nunes, Maria Antónia Duarte

**Affiliations:** 1 71001 Department of Gastroenterology, Hospital do Divino Espírito Santo de Ponta Delgada EPE, Ponta Delgada, Portugal; 2 Department of Gastrenterology, Hospital do Divino Espírito Santo de Ponta Delgada EPE, Ponta Delgada, Portugal

A 60-year-old man presented with dysphagia due to a 40-mm leiomyoma of the muscularis propria in the middle third of the esophagus. The patient had a history of hypertension, atrial fibrillation, and anticoagulation. Endoscopic submucosal tunneling resection (STER) was proposed ( Video 1 ).

Submucosal tunneling endoscopic resection of an esophageal leiomyoma.Video 1

An incision of the mucosa was performed 5 cm proximal to the lesion, creating a wide tunnel 4 cm above the lesion ( Fig. 1 ). The tunnel was created by separating the mucosa from the lesion, extending laterally and distally. Dissection of the tumor's attachment to the muscular layer was performed using the DualKnife J (Olympus, Tokyo, Japan) and the HybridKnife I (Erbe, Tuebingen, Germany) ( Fig. 2 ), and myotomy of the circular muscular layer (Triangle tip knife, Olympus)was performed, preserving the capsule ( Fig. 3 ). A retrieval net (Roth Net; Steris, Mentor, Ohio, USA) was used for extraction ( Fig. 4 ). Hemostasis was verified and topical gentamycin was applied. The mucosal incision was closed with clips.

**Fig. 1:**
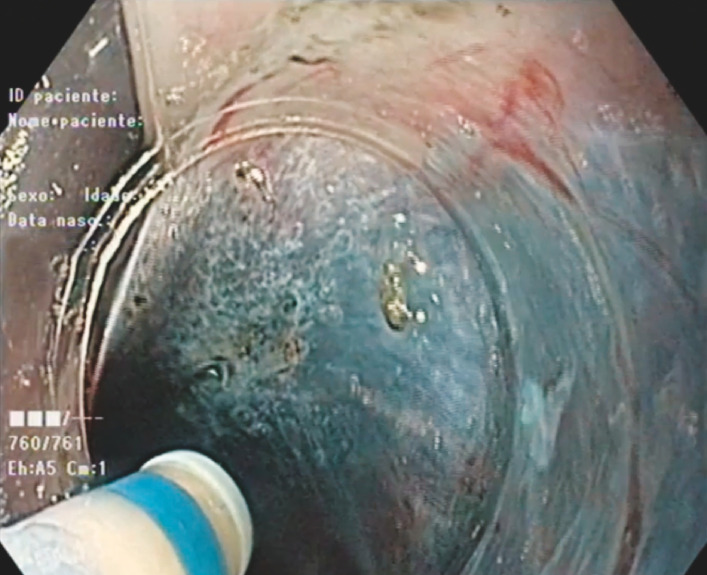
Tunnel 4 cm above the lesion, created by separating the mucosa from the lesion, extending laterally and distally.

**Fig. 2:**
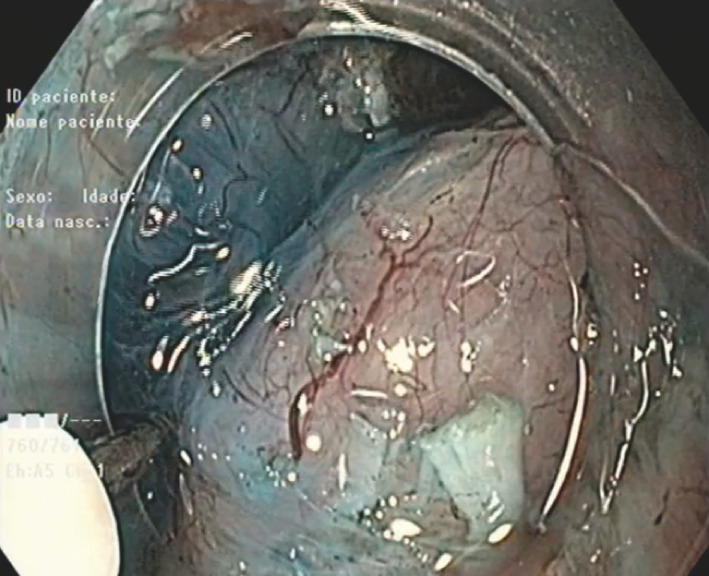
Dissection of the tumorʼs attachment to the muscular layer.

**Fig. 3:**
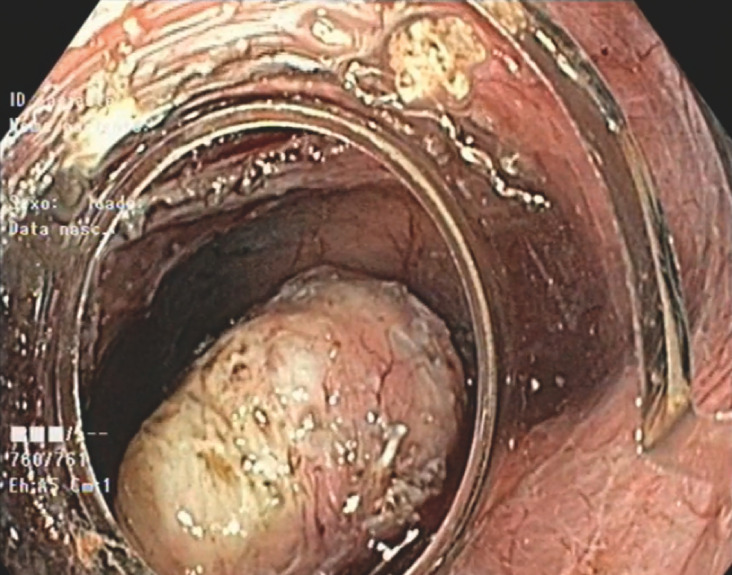
Capsule preservation with complete resection of the lesion.

**Fig. 4:**
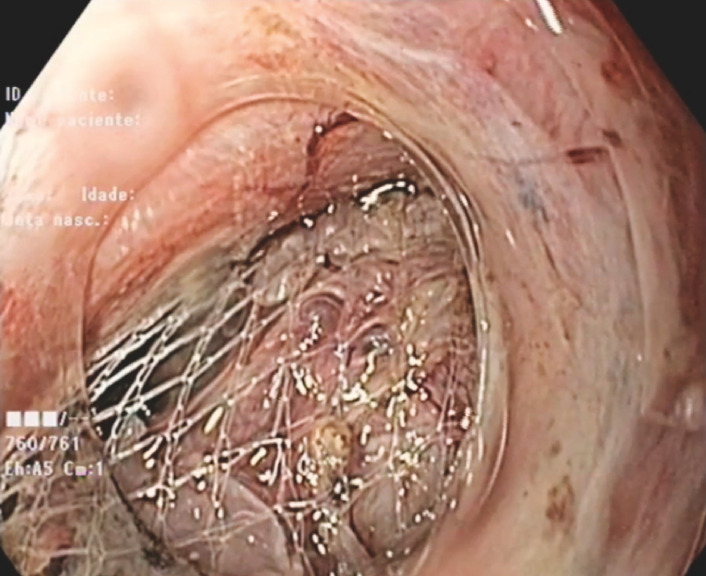
Extraction through the tunnel and the upper esophageal sphincter using a retrieval net.

The patient was admitted to an Intermediate Care Unit, with piperacillin-tazobactam antibiotic prophylaxis. After 24 hours, thoracalgia developed and a thoracoabdominal computed tomography scan revealed an esophageal hematoma. The patient made favorable progress with transfer to the ward after 72 hours and was discharged on the eighth day. Pathological diagnosis confirmation of esophageal leiomyoma was made ( Fig. 5 ).

**Fig. 5:**
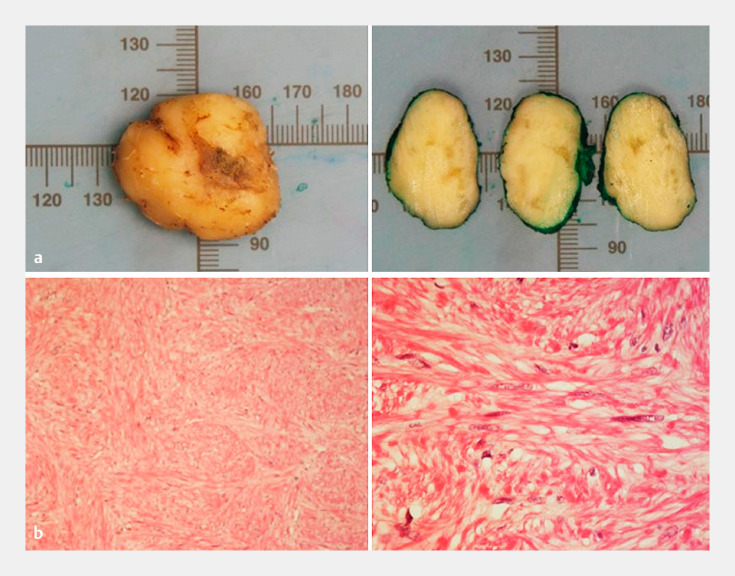
Histological evaluation. **a** White nodule of firm consistency. **b** Nodule formed by fusiform cells with no dysplasia (×20 magnification and ×100 magnification).

We present a case of a symptomatic esophageal leiomyoma successfully treated endoscopically with STER. Leiomyomas are benign lesions requiring resection when they cause obstructive symptoms, with an upper size limit for an endoscopic en bloc resection advised by the European guidelines of 35 mm, below that of the presented case 1 . STER allows the resection of tumors by tunneling the submucosa, minimizing the mucosal defect 2 3 . Thoracoscopy enucleation is a surgical alternative, although no comparative studies are available 4 . Endoscopic full-thickness resection may be less preferable to STER since it does not preserve mucosal integrity 3 . The development in third-space intervention enables the management of progressively more complex lesions, avoiding a major surgical intervention.

Endoscopy_UCTN_Code_TTT_1AO_2AG

## Notes

Endoscopy E-Videos https://eref.thieme.de/e-videos
*E-Videos* is an open access online section of the journal *Endoscopy* , reporting on interesting cases and new techniques in gastroenterological endoscopy. All papers include a high-quality video and are published with a Creative Commons CC-BY license. Endoscopy E-Videos qualify for HINARI discounts and waivers and eligibility is automatically checked during the submission process. We grant 100% waivers to articles whose corresponding authors are based in Group A countries and 50% waivers to those who are based in Group B countries as classified by Research4Life (see: https://www.research4life.org/access/eligibility/ ). This section has its own submission website at https://mc.manuscriptcentral.com/e-videos .
